# Differential attainment in undergraduate medical education: a systematic review

**DOI:** 10.1192/bjo.2021.128

**Published:** 2021-06-18

**Authors:** Abhishek Gupta, Shreya Varma, Radhika Gulati, Natasha Rishi, Nagina Khan, Rohit Shankar, Subodh Dave

**Affiliations:** 1 University of Birmingham, Medical School; 2 Queen's University Belfast; 3 South London and Maudsley NHS Foundation Trust; 4 Touro University Nevada; 5 Exeter Medical School, Cornwall Partnership NHS Foundation Trust, Chair Royal College of Psychiatrists South West Division; 6 University of Bolton, Consultant Psychiatrist, Derbyshire Healthcare Foundation Trust; Hon. Asso. Professor, University of Nottingham, Dean Elect, Royal College of Psychiatrists

## Abstract

**Aims:**

Differential attainment (DA) amongst Black and Minority Ethnic (BAME) medical students and postgraduate trainees including Psychiatry trainees has been extensively documented in medical education, with non-white medical students being 2.5 times more likely to fail high-stake examinations compared to their White counterparts. The Equality Act 2010 places a responsibility on public bodies such as Royal Colleges to address discrimination in training and assessment. Understanding DA in undergraduate medical education can help understand DA in the postgraduate setting. Consequently, this systematic review aims to detect the processes that enable and impede DA in UK undergraduate medical education.

**Method:**

Seven online databases including PubMed, Scopus, PyschInfo, and ERIC were searched. A formal grey literature search was also conducted. Inclusion criteria comprised studies dated from January 1995 to present and included UK undergraduate medical students. We present the preliminary findings from 13 papers, analysed to create a conceptual framework for a further mixed methods analysis. The studies were critically appraised for methodological quality.

**Result:**

Five key themes emerged from the preliminary analysis of 13 papers. BAME students experienced:

Being ‘divergent’: Not feeling part of the current organisational learning milieu

Lack of social capital: Difficulty in being absorbed into existing ‘networks’ of relationships in a manner that is ‘approachable’ and not ‘intimidating’

Continuum of discrimination: ‘Indirect’ impact of subtle communication processes in the learning environment undermining individual ‘belief’ in own performance

Institutional discriminatory factors: Culture, rules, norms, and behavioural routines of educators that lead to differential outcomes for learners

Lack of external support: Relative lack of interventions tackling DA.

**Conclusion:**

The key finding of this review is that British BAME undergraduate medical students experience discriminatory behaviours early in medical schools that impact on personal, educational, and professional outcomes. These factors may need to be borne in mind by postgraduate training organisations such as the Royal College of Psychiatrists as they commence the challenging task of addressing DA.

